# Ubiquitin ligases join the field of dietary restriction in *C.elegans*

**DOI:** 10.18632/aging.100084

**Published:** 2009-09-03

**Authors:** Mark Lucanic, Pankaj Kapahi

**Affiliations:** Buck Institute for Age Research, Novato, CA 94945, USA

**Keywords:** C elegans, dietary restriction, ubiquitin ligases

Dietary restriction (DR) has been shown to robustly modulate organismal lifespan in multiple species. The powerful genetic tools in yeast, worms and flies hold great promise in unraveling the secrets of the molecular mechanisms of this process that have been elusive for more than seven decades, since DR was first described to extend lifespan in mice. In a recent study, Carrano and colleagues report the discovery that an E2/E3 ubiquitin ligase set are required for the lifespan extending effects of DR in *C. elegans* [1].

The authors utilize *C. elegans* to look at E3 ubiquitin ligases of the conserved HECT (Homologous to E6-AP Carboxyl Terminus) family known to be important in tumorigenesis in mammals [2]. *C. elegans* contains a single ortholog of the WW class of HECT E3s known as WWP-1. Mutant analysis revealed that the *C. elegans* gene is required for embryonic development but that weak mutants or naive adults exposed to *wwp-1* dsRNA have wildtype lifespan under normal culture conditions but are sensitive to heat and oxidative stress. Stress response genes are often involved in modulating normal lifespan in a positive manner. Consistent with this notion the authors find that overexpression of *wwp-1* leads to a lengthened lifespan in otherwise wildtype animals. As these results implicate WWP-1 as having both a stress resistance and pro-longevity function the authors next test whether the gene contributes to other known longevity pathways.

Insulin/IGF signaling, mitochondrial function and nutrients control distinct aspects of *C. elegans* lifespan. To test whether WWP-1 functions in any of these longevity pathways, the authors examine mutant combinations with essential components of these pathways. They find that a loss of *wwp-1* in long lived insulin/IGF and mitochondrial mutants results in a modest shortening of their extended lifespan. However, the lifespan extension in *eat-2* mutants, which eat less due to reduced pharyngeal pumping rate and are therefore under DR, is abrogated. They also convincingly show that WWP-1 is involved in DR by using a method that utilizes bacterial dilution to impart lifespan extension. One of the dramatic findings was that the 3 fold extension of lifespan achieved by reducing food concentration in liquid culture is almost entirely suppressed by a loss of *wwp-1*. These experiments genetically implicate WWP-1 as being a key player in the DR pathways of longevity.

WWP-1 is orthologous to mammalian E3 ubiquitin ligases and the authors show biochemically, that WWP-1 also has E3 activity *in vitro*. Disruption of a key residue in the catalytic domain abolished both the *in vitro* activity and its ability to rescue the DR phenotype. As part of the ubiquitin targeting process E3s function in a cascade that has  immediately upstream of it an E2 ubiquitin conjugating enzyme, WWP-1 had previously been found to interact with a specific E2 ubiquitin conjugating enzyme known as UBC-18 [3]. The authors find from their *in vitro* assay that most of the functional E3 activity of WWP-1 is dependent on the presence of UBC-18. Furthermore, they show that the two proteins (UBC-18 and WWP-1) interact in a GST pull down assay. Finally the authors show that *ubc-18* behaves genetically similar to *wwp-1* in that it also is required for DR induced lifespan extension.

An understanding of the molecular pathways that mediate the protective effects of DR is one of the most important questions in biomedicine. Previous studies in *C. elegans* have identified novel genetic pathways that determine the beneficial effects of DR [4,5,6,7,8]. Key regulators of DR-dependent lifespan extension include transcription factors and environmental sensors such as PHA-4, SKN-1, HSF-1, and AMPK, which were identified by different dietary regimens that restrict nutrient intake. TOR (Target of Rapamycin) is also emerging as a key regulator in mediating the effects of DR and is conserved across species. It is has been shown to modulate lifespan extension in flies [9], yeast [10], worms [11] and most recently mice [12]. The signaling pathways that mediate the effects of DR are complex and impinge on a variety of cellular targets. For example, the TOR pathway was recently shown to modulate the effects of DR in *C. elegans* through HIF-1 acting downstream of S6K to modulate DR-dependent lifespan extension via the IRE-1 ER stress pathway [13]. It is now becoming apparent that the maximal lifespan extension by DR is not achieved by regulating a single genetic pathway but that multiple pathways act together to mediate the lifespan effect of DR. Future research examining how the E2/E3 ligase set identified by Carrano and colleagues interacts with other pathways of DR in *C. elegans* will be of great interest. Importantly the E2/E3 ligase set likely functions to degrade or modulate a target protein(s) whose non-ubiquitinated function shortens lifespan in stressful conditions such as low nutrient availability and high temperature. Identification of the upstream signaling cascades that activate this E2/E3 ligase set and their specific targets should yield a better understanding of how animals modulate their lifespan based on environmental conditions such as nutrient availability.
